# A Rare Infectious Complication Following Transrectal Prostate Biopsy: Spondylodiscitis With Contiguous Psoas Muscle Abscess

**DOI:** 10.7759/cureus.102560

**Published:** 2026-01-29

**Authors:** Luis Carlos, Ronaldo Mayta, Mijail Vega, Diego Salazar, Andrea Sanchez

**Affiliations:** 1 Faculty of Medicine, Universidad Privada San Juan Bautista, Ica, PER; 2 Faculty of Medicine, Universidad Católica de Santa María, Arequipa, PER; 3 Faculty of Medicine, Universidad Peruana de Ciencias Aplicadas, Lima, PER; 4 Faculty of Medicine, Universidad Autónoma del Estado de Hidalgo, Pachuca, MEX

**Keywords:** escherichia coli, image-guided drainage, infectious spondylodiscitis, magnetic resonance imaging, psoas abscess, transrectal prostate biopsy

## Abstract

Spondylodiscitis is a rare but potentially serious spinal infection. Although *Staphylococcus aureus* is the most common pathogen, *Escherichia coli* has been increasingly associated with genitourinary procedures such as transrectal prostate biopsy, even with antibiotic prophylaxis.

We present the case of a 71-year-old man who underwent a transrectal prostate biopsy due to elevated prostate-specific antigen (PSA) levels. He developed fever and lower back pain 24 hours after the procedure. Initial studies suggested a bacterial infection, and empirical antibiotic treatment was initiated. Lumbar magnetic resonance imaging (MRI) revealed L4-L5 spondylodiscitis with left paravertebral involvement and an ipsilateral psoas abscess. Computed tomography (CT)-guided percutaneous drainage and disc biopsy were performed. Cultures isolated *E. coli*, allowing for adjustment of the antibiotic treatment, with a favorable clinical outcome.

Although spondylodiscitis secondary to transrectal prostate biopsy is uncommon, the presence of fever and lower back pain was key to the diagnostic suspicion. Imaging studies, particularly MRI, proved essential for establishing the diagnosis and guiding effective treatment.

Infectious spondylodiscitis is a rare but potentially fatal complication following transrectal prostate biopsy. Early clinical suspicion and prompt pathogen identification are essential. MRI and image-guided minimally invasive procedures, including percutaneous biopsy and drainage, play a key role in diagnosis and effective management.

## Introduction

Spondylodiscitis is an infectious inflammation of the intervertebral discs, vertebral bodies, and adjacent structures secondary to an infectious agent, primarily through hematogenous dissemination [1]. Although it has a very low incidence of seven cases per million inhabitants, if not detected in time, it can be potentially fatal, due to the possibility of vertebral osteomyelitis with progressive bone destruction, abscess formation, and severe neurological compromise [2]. The majority of reported cases are due to *Staphylococcus aureus*, but gram-negative bacilli such as *Escherichia coli* are also reported in patients with urinary tract infections or urological procedures [3].

Transrectal prostate biopsy is one of the most commonly used procedures today for diagnosing prostate cancer. While it is considered a safe procedure when ultrasound-guided, minor complications such as urinary retention, hematuria, and rectal bleeding are common. Infectious complications may go unnoticed due to the antibiotic prophylaxis received by patients undergoing this procedure, as in our case [4].

Here, we present a rare case of a 71-year-old male patient who underwent a transrectal prostate biopsy procedure and experienced two major infectious complications: spondylodiscitis with paravertebral abscess and abscess of the left psoas muscle caused by gram-negative bacilli (*E. coli*) despite prophylactic treatment with fluoroquinolones.

## Case presentation

A 71-year-old man initially presented to the urology outpatient clinic of Hospital Félix Torrealva Gutiérrez, a public hospital affiliated with EsSalud in Ica, Peru, with a prostate-specific antigen (PSA) level of 7 ng/mL (reference range: <4 ng/mL) and lower urinary tract symptoms (LUTS) that were improving with tamsulosin 0.4 mg, one tablet every 24 hours. Based on this laboratory result and a relevant physical examination, which revealed a 5x5 cm, fibroelastic, mobile prostate with well-defined borders and no nodules, the urologist recommended a transrectal prostate biopsy. As part of standard management, antibiotic prophylaxis with fluoroquinolones (ciprofloxacin 500 mg every 12 hours for 10 days) was prescribed, with instructions to begin treatment three days prior to the procedure.

After 24 hours of the procedure, the patient went to the emergency department because of an intermittent, unquantified sensation of fever, with a temperature of 38.4°C found on physical examination. During this visit, an initial blood count was performed, which showed normocytic normochromic anemia and marked relative neutrophilia with severe lymphopenia and mild thrombocytopenia, hematological findings suggestive of an early inflammatory response, even in the absence of leukocytosis (Table 1).

**Table 1:** Main laboratory findings in the blood count upon admission by the emergency department. VCM, mean corpuscular volume; HCM, mean corpuscular hemoglobin; CHCM, mean corpuscular hemoglobin concentration.

Type	Value	Unit	Reference Range
Hemoglobin assay	11.5	g/dL	13-17
Hematocrit	34.3	%	40-50
VCM	90.1	fL	80-100
HCM	30.2	pg	27-33
CHCM	33.5	g/dL	32-36
Total leukocytes	5.04	×10³/µL	4.0-11.0
Neutrophils	95.1	%	40-75
Lymphocytes	3.9	%	20-45
Platelets	126	×10³/µL	150-450

**Figure 1:**
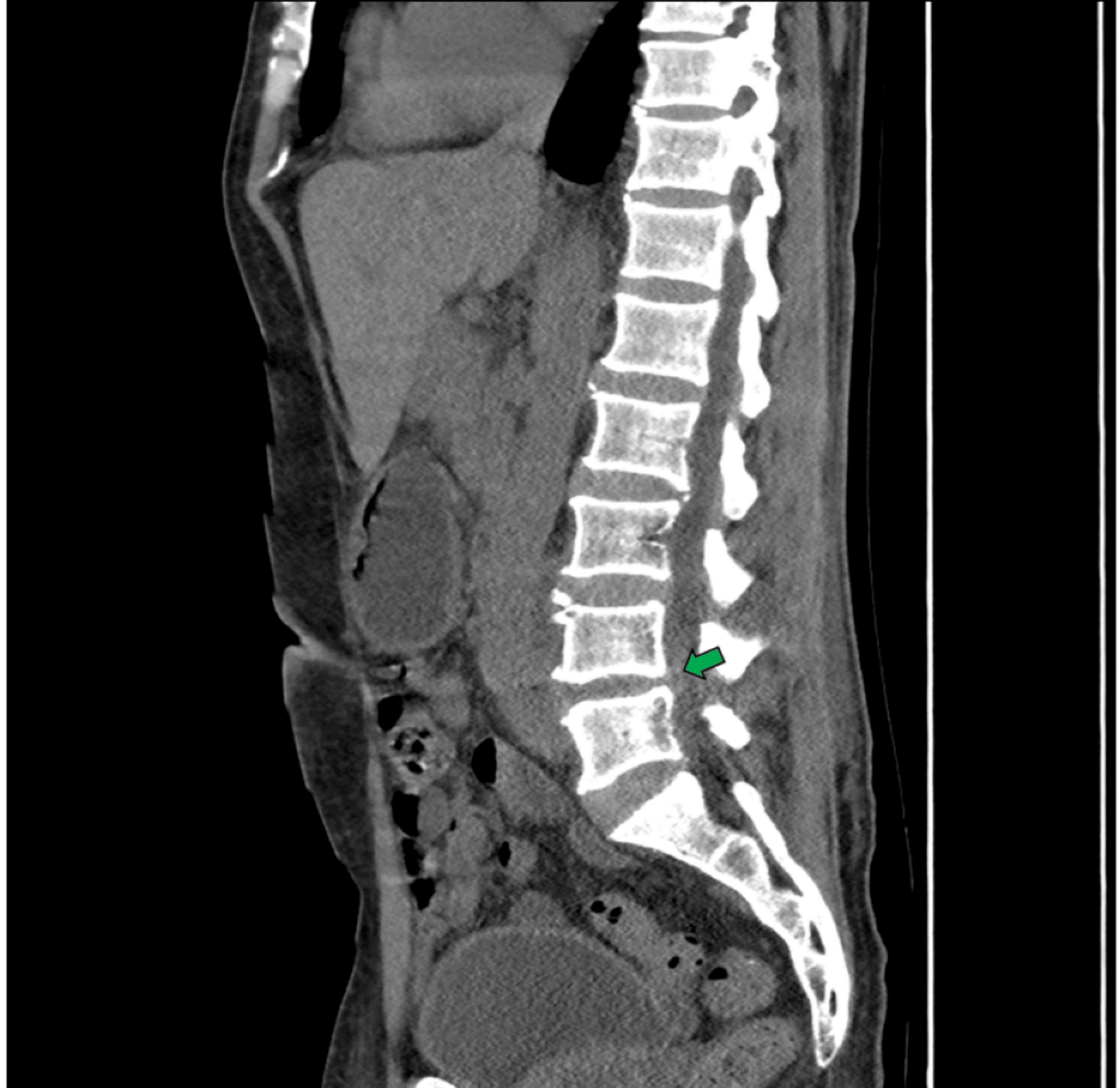
Abdominopelvic CT scan, sagittal view. The green arrow indicates degenerative changes in the lumbar spine, characterized by deforming spondylosis and L4-L5 intervertebral osteochondrosis. CT, computed tomography.

This patient was hospitalized in the internal medicine service, where empirical broad-spectrum antibiotic treatment with carbapenems (Imipenem 500 mg IV every 6 hours for six days) was initiated due to suspected severe infection associated with a urological procedure and the possibility of resistant gram-negative bacilli. However, carbapenems are considered last-line agents for severe infections caused by resistant gram-negative organisms, and their use may promote the emergence of antimicrobial resistance if not judiciously indicated. During hospitalization, the patient developed progressive lower back pain, so an abdominopelvic computed tomography (CT) scan was ordered, which revealed degenerative findings, such as marginal osteophytes and narrowing of the L4-L5 intervertebral space with sclerosis of the endplate (Figure 1).

**Figure 2:**
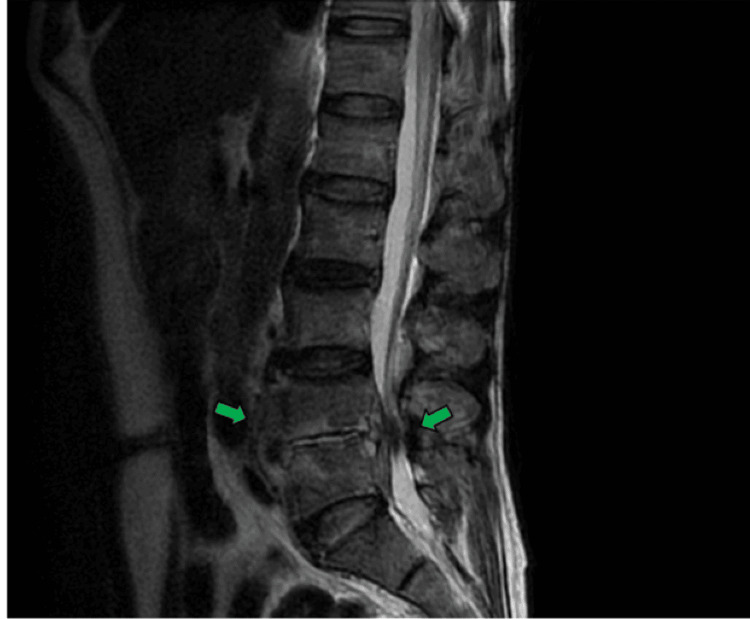
Contrast-enhanced MRI of the lumbar spine, sagittal view, T2 FRFSE sequence. The green arrows indicate signs of spondylodiscitis at the L4-L5 level, showing bone edema of the adjacent vertebral bodies and left paravertebral inflammatory involvement. T2 FRFSE, T2 fast recovery fast spin echo.

**Figure 3:**
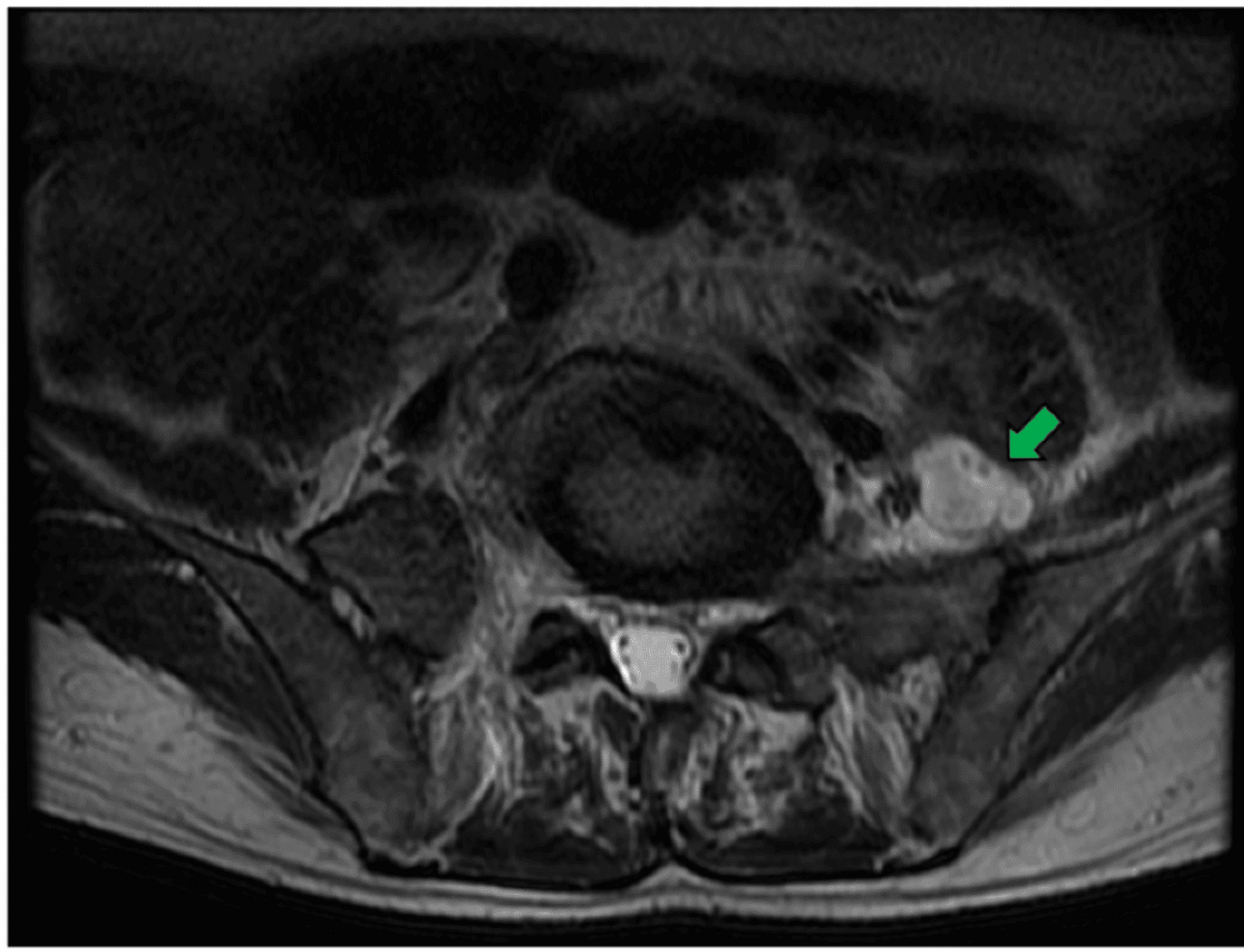
Contrast-enhanced MRI of the lumbar spine, axial view, T2 FRFSE sequence. The green arrow indicates a hyperintense collection consistent with an abscess in the left psoas muscle. MRI, magnetic resonance imaging; T2 FRFSE, T2 fast recovery fast spin echo.

Due to the lack of clinical improvement, the patient was referred to Hospital IV Augusto Hernández Mendoza, a tertiary-care center, where a contrast-enhanced lumbar spine magnetic resonance imaging (MRI) was performed. This revealed signs of spondylodiscitis at the L4-L5 level, including bone edema of adjacent vertebral bodies and left paravertebral inflammation (Figure 2), as well as a 10 cc abscess in the left psoas muscle (Figure 3). While awaiting definitive etiological studies, empirical intravenous antibiotic treatment with ciprofloxacin and metronidazole was restarted.

**Figure 4:**
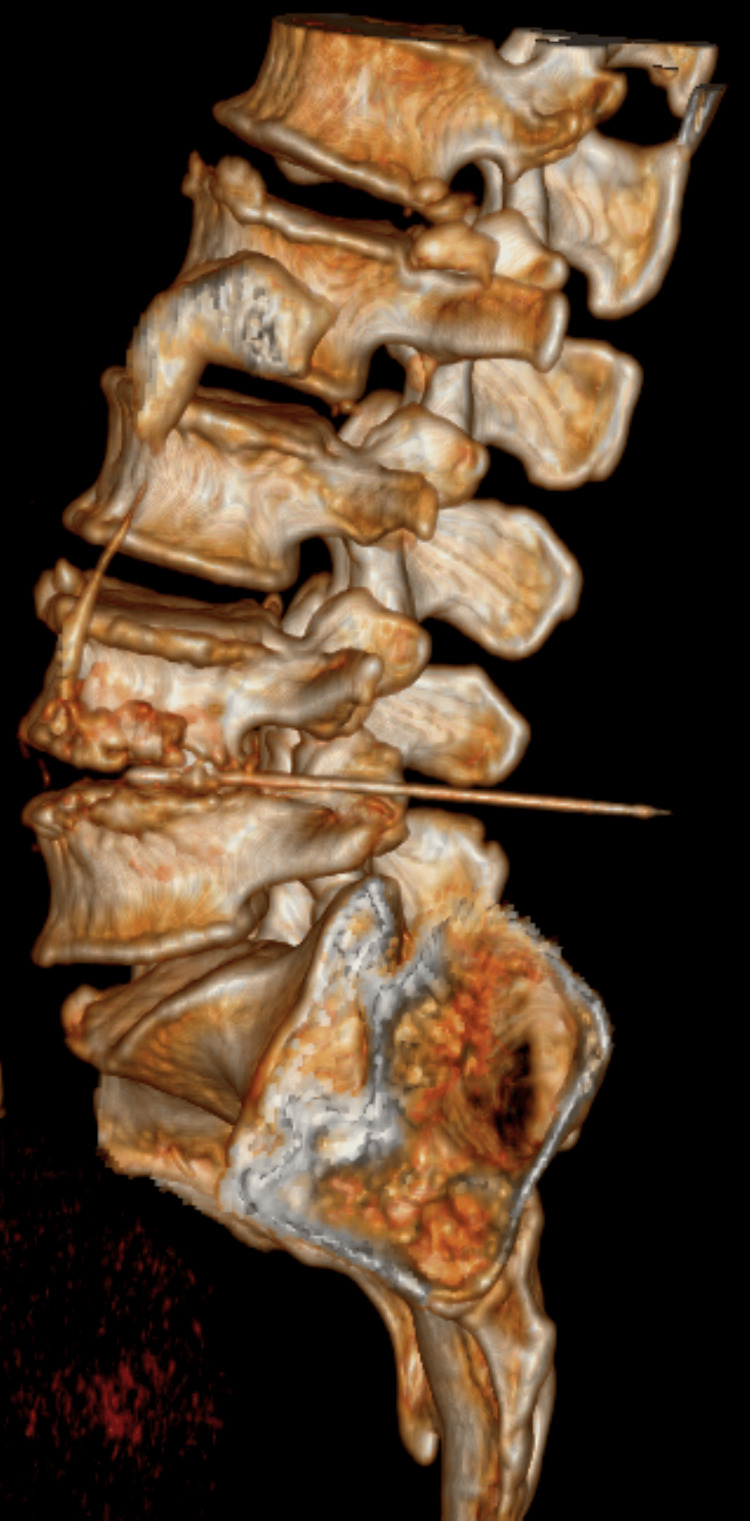
CT of the lumbar spine with 3D sagittal reconstruction, showing CT-guided percutaneous biopsy of the intervertebral disc (L4-L5), performed using a Tru-Cut needle with a coaxial system. CT, computed tomography; 3D, three-dimensional.

Given the persistence of the clinical picture and the identification of an abscess in the left psoas, the patient was evaluated by the interventional radiology service, where a percutaneous drainage guided by tomography of the collection of the left psoas was performed with a 22G chiba needle; in addition, a tomography-guided biopsy of the intervertebral disc (L4-L5) and adjacent vertebral bodies was performed using a Tru-Cut needle with coaxial system (Figure 4), obtained samples for culture, and sent for reading by pathology.

**Figure 5:**
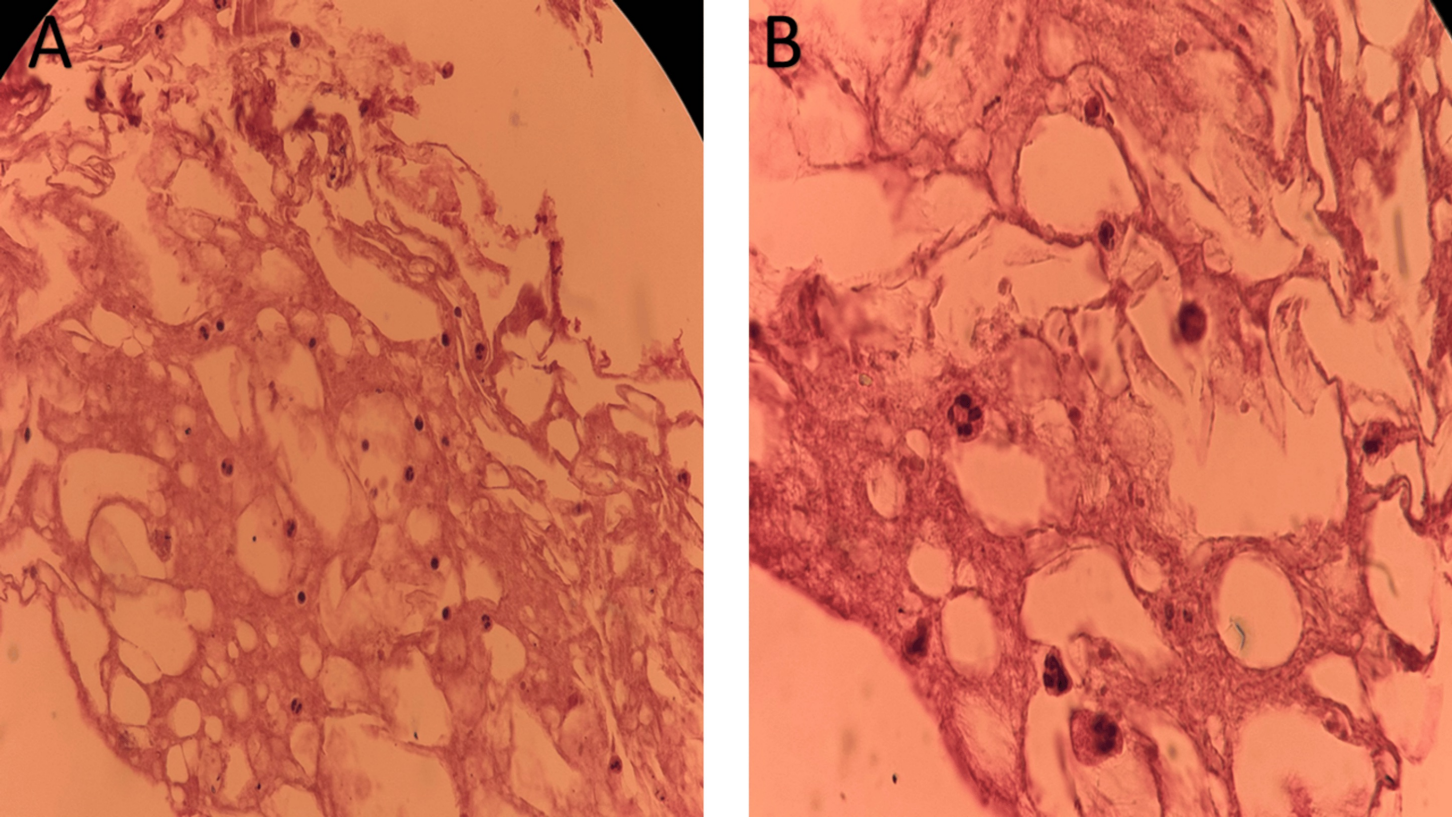
Histopathological findings from L4–L5 intervertebral disc biopsy. (A) Low-power photomicrograph showing fibroconnective tissue with areas of fat necrosis and stromal hyalinization. (B) Higher magnification demonstrating mild inflammatory infiltrate composed of polymorphonuclear leukocytes and lymphocytes, without evidence of malignancy (hematoxylin and eosin stain).

Microbiological analysis of both the intervertebral disc biopsy and the drained psoas abscess revealed the growth of *E. coli*. Gram staining was not performed; however, culture results showed no evidence of polymicrobial infection. In addition, the isolated germ showed resistance to fluoroquinolones and trimethoprim-sulfamethoxazole, while remaining sensitive to beta-lactams and carbapenems (Table 2). Histopathological examination revealed fibroconnective and muscular tissue with areas of fat necrosis, hemorrhage, and a mild inflammatory infiltrate, without evidence of malignancy (Figure 5A, 5B).

**Table 2:** Microbiological findings from intervertebral disc biopsy and psoas abscess. Culture from both the intervertebral disc biopsy and drained psoas abscess grew *Escherichia coli*, showing resistance to fluoroquinolones and trimethoprim-sulfamethoxazole, while remaining sensitive to beta-lactams and carbapenems.

Study	Result
Culture (disc biopsy)	Escherichia coli
Culture (psoas abscess)	Escherichia coli
Fluoroquinolones	Resistant
Trimethoprim–sulfamethoxazole	Resistant
Beta-lactams	Sensitive
Carbapenems	Sensitive

Finally, after clinical improvement, control of the focus of infection through percutaneous drainage, and microbiological identification of the causative agent, the antimicrobial treatment was adjusted. Considering the sensitivity of the isolated *E. coli* to beta-lactams, the patient's clinical stability, the absence of fever, adequate tolerance of oral medication, and the need to complete a prolonged course of treatment, the patient was discharged with oral amoxicillin-clavulanic acid (500/125 mg every 8 hours), with a planned total antibiotic treatment of eight weeks. In subsequent follow-up visits, the complete blood count showed normalization of parameters, consistent with the favorable clinical evaluation.

## Discussion

The case of a 71-year-old man was presented, who, after undergoing a transrectal prostate biopsy, presented an infectious complication with fluoroquinolone-resistant *E. coli* as the etiological agent.

Spondylodiscitis has a low incidence, being more common in men than in women [2]. It is more common in people between the ages of 60 and 80 [5]. As described in the literature, transrectal prostate biopsies have a higher risk of infection than other approaches [6,7]. The most common pathogens in this infection are *Staphylococcus aureus* and *E. coli* [2]. This case is consistent with the recorded epidemiology, as the patient is male and falls within the recorded age group. The infection present in this case reaffirms the risk of infection associated with transrectal prostate biopsy procedures.

The clinical presentation consists of a combination of spinal syndrome and infectious syndrome, although fever is usually absent in 50% of patients [8]. In this case, the patient presented with fever within the first 24 hours, accompanied by chills, followed by lower back pain. According to the literature, spinal symptoms are the most frequent symptom, present in 85-90% of patients, and usually manifest at the onset of the clinical picture, characterized by an insidious onset and worsening with movement [9]. In our patient, the early presence of spinal symptoms was key to guiding the diagnostic suspicion and requesting auxiliary tests.

In laboratory tests, several studies report that elevated C-reactive protein (CRP), erythrocyte sedimentation rate (ESR), and leukocytosis are common but non-specific markers in cases of spondylodiscitis [10]. In our patient, laboratory findings demonstrated leukocytosis with marked neutrophilia, which supported the clinical suspicion of an underlying infectious process and correlated with previously reported laboratory patterns in the literature. Although these markers are not diagnostic on their own, they contributed to the overall clinical assessment and reinforced the need for further imaging studies.

It has been shown that percutaneous biopsy is necessary when a rare germ is suspected, or the cause is not evident [11]. A CT scan guided by a specific imaging technique is very helpful for performing this procedure, and that is what was done in our patient. While the CT scan demonstrates osteolysis, erosions, and other signs suggestive of an abscess, it does not clearly show their extent or severity. In contrast, MRI is the gold standard for detecting spondylodiscitis [12]. This contrast-enhanced lumbar spine imaging study was the most helpful diagnostic test in this case.

Treatment usually lasts 4-12 weeks and should be guided by microbiological culture and antimicrobial susceptibility testing [2]. In our patient, fluoroquinolone prophylaxis was administered prior to prostate biopsy; however, he subsequently developed a severe infectious complication, suggesting either inadequate coverage or infection by a resistant organism. For this reason, empirical intravenous imipenem was initiated due to concern for a complicated infection caused by resistant gram-negative bacilli. As clinical response remained poor, a second empirical regimen with intravenous ciprofloxacin and metronidazole was started to broaden coverage for possible mixed aerobic and anaerobic organisms. Despite this, the patient showed no clinical improvement. Following CT-guided percutaneous biopsy, the causative pathogen was identified as fluoroquinolone-resistant *E. coli* with preserved susceptibility to beta-lactams. Consequently, antibiotic therapy was de-escalated to targeted oral amoxicillin/clavulanic acid (500/125 mg every 8 hours), with a planned total treatment duration of eight weeks.

Infections are the most common complication of transrectal prostate biopsy and the most common cause of hospitalization [13]. Infection of the psoas-iliac muscle usually corresponds to a secondary process due to dissemination from adjacent structures, with a predominance of vertebral foci, compared to urological procedures, as mentioned by Cheng Xu et al., where 11 of 20 cases were due to infection secondary to spondylodiscitis and spondylitis [14]. The complications that the patient experienced are extremely rare and seldom reported.

## Conclusions

Infectious spondylodiscitis is a rare but potentially life-threatening complication following transrectal prostate biopsy, even in patients who receive antibiotic prophylaxis. This case underscores the importance of maintaining early clinical suspicion when symptoms are nonspecific and laboratory findings are inconclusive. MRI remains the diagnostic modality of choice for the timely detection of vertebral involvement and associated complications. Image-guided percutaneous biopsy and drainage played a crucial role in identifying the causative pathogen and guiding targeted antimicrobial therapy. This report highlights the need for close monitoring after urological procedures and emphasizes the value of interventional radiology in early diagnosis and minimally invasive management of atypical infectious complications.
